# Human gingival fibroblast-mediated remodeling of three-dimensional fibrin hydrogels

**DOI:** 10.1016/j.mbplus.2026.100191

**Published:** 2026-02-07

**Authors:** Merve Ceylan, Marja L. Laine, Behrouz Zandieh Doulabi, Hans J.A.M. Korfage, René J.B. Dijkstra, Teun J. de Vries, Ton Schoenmaker

**Affiliations:** a Department of Periodontology, Academic Centre for Dentistry Amsterdam, University of Amsterdam and Vrije University Amsterdam, Amsterdam, the Netherlands; b Department of Oral Cell Biology, Academic Centre for Dentistry Amsterdam, University of Amsterdam and Vrije University Amsterdam, Amsterdam, the Netherlands; c Department of Orofacial Anatomy, Academic Centre for Dentistry Amsterdam, University of Amsterdam and Vrije University Amsterdam, Amsterdam, the Netherlands; d Center for Dentistry and Oral Hygiene, University Medical Center Groningen, University of Groningen, Groningen, the Netherlands

**Keywords:** Gingival fibroblasts, Fibrin, 3D cell culture, Extracellular matrix, Wound healing

## Abstract

The extracellular matrix (ECM) critically regulates fibroblast behavior during tissue repair and regeneration. However, how culture dimensionality influences fibroblast-mediated ECM remodeling remains unclear. This study investigated the effects of three-dimensional (3D) fibrin hydrogels on the phenotype and remodeling activity of primary human gingival fibroblasts (GFs) compared to conventional two-dimensional (2D) monolayer cultures. Live/dead staining confirmed high GF viability in both conditions, with elongated and branched cell morphologies in 3D fibrin hydrogels, contrasting with spindle-shaped cells in 2D monolayers. Hematoxylin and Eosin, and Masson’s Trichrome staining revealed progressive fibrin degradation and *de novo* collagen deposition over 21 days of culturing. Gene expression analysis showed that while *FN1*, *COL1A1*, and *COL3A1* levels remained relatively stable, *TGFB1* expression increased significantly from day 7 to day 14 in 3D hydrogels (*p* < 0.05) and was higher than in 2D cultures at day 21 (*p* < 0.05). This coincided with a marked upregulation of *ACTA2* (*p* < 0.01), indicating myofibroblast-like differentiation. MMP-2 activity increased significantly over time in both 2D and 3D cultures (*p* < 0.01 and *p* < 0.001, respectively). In contrast, *PLAU* and *PLAT* expression decreased significantly at days 14 and 21 (*p* < 0.001 and *p* < 0.05, respectively), reflecting a temporal shift from fibrinolytic to collagenolytic remodeling. Despite active remodeling, mechanical testing showed no significant changes in hydrogel stiffness or relaxation between day 1 and day 7, or between cell-seeded and acellular gels (*p* > 0.05), likely due to the small contractile forces generated by the cells relative to the gel’s bulk modulus. Together, these findings demonstrate that 3D fibrin hydrogels provide a biologically active and physiologically relevant microenvironment that supports fibroblast-mediated ECM remodeling, offering a biomimetic model for investigating the mechanobiology of periodontal and *peri*-implant soft tissue regeneration.

## Highlights

• 3D fibrin hydrogels promote in vivo-like morphology in gingival fibroblasts.
• Gingival fibroblasts remodel fibrin matrices and deposit newly synthesized collagen.
• Decreased u-PA/t-PA and increased MMP-2 indicate a shift to collagenolytic remodeling.
• Fibrin hydrogel stiffness remains stable despite active matrix remodeling.
• 3D fibrin matrices provide a biomimetic model for periodontal soft tissue regeneration.

## Introduction

The periodontium is a specialized tissue complex that supports and anchors the teeth. It comprises the gingiva, periodontal ligament, alveolar bone, and cementum [1]. In chronic inflammatory conditions such as periodontitis, this system undergoes progressive structural degradation, ultimately resulting in the loss of tooth-supporting tissues [1]. Recent evidence has identified fibrin deposition as a defining feature in the gingiva adjacent to sites of alveolar bone degradation in periodontitis, but not in periodontally healthy tissues, suggesting that fibrin may play a regulatory role in inflammation, wound healing, or bone regeneration [2]. Effective regeneration of the lost tissue within the periodontium is therefore essential for maintaining oral health and preventing further tissue breakdown [3], [4]. Conventional periodontal therapies inevitably cause wounding of the already inflamed tissues, and their outcomes largely depend on the cellular and molecular events that orchestrate wound healing [5]. A deeper understanding of the underlying cellular and molecular mechanisms of periodontal wound healing is critical for the development of targeted therapeutic interventions [3], [4].

Gingival fibroblasts (GFs), the predominant cell type within the gingival connective tissue, play a pivotal role in coordinating wound healing responses [6], [7]. These cells are responsible for the synthesis, deposition, and remodeling of the extracellular matrix (ECM), and they actively respond to biochemical and biomechanical cues within their microenvironment [6], [7]. GFs are characterized by their high plasticity, capacity for scarless healing, and ease of isolation, all of which render them particularly advantageous for use in studying wound healing and potential therapeutic applications [8], [9].

One of the earliest events in wound healing is the formation of a provisional ECM primarily composed of fibrin, which is generated through thrombin-mediated polymerization of fibrinogen [4], [10]. Fibroblasts are subsequently recruited to the wound site, where they contribute to granulation tissue formation, ECM synthesis, and tissue remodeling[10]. This fibrin matrix not only facilitates hemostasis but also provides a transient three-dimensional (3D) scaffold that supports cellular infiltration, adhesion, proliferation, and migration during the initial stages of wound healing [11], [12]. The fibrin forms a 3D hydrogel structure that supports surrounding cells and promotes cell proliferation and migration [11], [13]. San Martin et al. previously demonstrated that fibrin scaffolds enhance oral fibroblast proliferation and stimulate the synthesis of ECM components, including type I and III collagen, thereby, underscoring their relevance as a biomimetic substrate [14]. However, the dynamic mechanisms by which fibroblasts remodel the fibrin matrix through coordinated regulation of proteolytic activity, gene expression, and cell-matrix signaling, remain incompletely understood.

Moreover, unlike synthetic hydrogels, fibrin has more functions than just acting as a passive structural matrix, it selectively binds a range of proteins and growth factors including fibronectin, vitronectin, fibroblast growth factor (FGF), vascular endothelial growth factor (VEGF), and enzymes like plasminogen and tissue-type plasminogen activator (t-PA) [11], [15]. In addition to its biological activity, fibrin is biocompatible, biodegradable, and allows easy cell migration due to its porous structure, all of which make fibrin a promising material for tissue engineering applications [11], [12], [16].

The composition and mechanical properties of fibrin can further influence fibroblast behavior, ultimately affecting overall wound-healing outcomes. For instance, Jansen et al. showed that fibroblast morphology and spreading were modulated by matrix stiffness [17]. In softer matrices with lower fibrin concentrations, fibroblasts exhibited pronounced elongated morphologies with dendritic-like protrusions, whereas stiffer matrices restricted cell spreading. They also reported that fibroblasts modified the stiffness of fibrin matrices by generating traction forces on fibrin matrices during spreading [17]. Importantly, in fibrin-based matrices, stiffness does not represent an isolated mechanical parameter. Unlike synthetic hydrogels, where matrix stiffness and ligand density can be independently tuned, increases in fibrin stiffness are typically achieved by elevating fibrinogen concentration. This approach simultaneously affects fibrin fiber density, network architecture, and the availability of cell-adhesive ligands. As a result, cellular responses reported in stiffer fibrin matrices reflect a combined influence of mechanical resistance, biochemical signaling, and microstructural organization rather than stiffness alone. This intrinsic coupling is a fundamental characteristic of fibrin networks and should be taken into account when interpreting stiffness-dependent cellular behaviors in fibrin-based culture systems [15], [18], [19].

*In vitro* models that recapitulate aspects of the wound healing microenvironment are essential for elucidating fibroblast behavior under defined conditions. A key parameter in such models is the dimensionality of the culture system. Traditional two-dimensional (2D) monolayer cultures fail to capture the complexity of the native extracellular milieu, whereas 3D culture platforms, such as fibrin hydrogels, provide a more physiologically relevant context by mimicking the spatial and mechanical characteristics of *in vivo* tissue [20], [21]. Previous studies have shown that culture dimensionality can markedly influence cell morphology, gene expression, and ECM remodeling in various cell types, including vascular smooth muscle cells (VSMCs) and lung fibroblasts [21], [22]. For instance, Hong *et al.* showed that VSMCs cultured in 3D fibrin or collagen matrices exhibited upregulated expression of ECM-related genes, including collagen I and fibronectin, compared to 2D cultures [21]. Conversely, Htwe *et al.* found that lung fibroblasts in 2D systems displayed exaggerated inflammatory signaling, whereas 3D cultures more accurately reflected *in vivo* cytokine responses [22].

Despite the increasing utilization of fibrin-based matrices in tissue engineering and wound healing research, direct comparative analyses of fibroblast behavior in 2D versus 3D culture environments remain relatively scarce, particularly in relation to periodontal soft tissue regeneration. Understanding how dimensionality modulates fibroblast-mediated ECM remodeling is essential for optimizing clinical applications, including the widely used fibrin derivatives leukocyte- and platelet-rich fibrin (L-PRF) and advanced PRF (A-PRF), which combine fibrin matrices with leukocytes [23].

We, therefore, aimed to determine how culture dimensionality influences fibroblast-mediated ECM remodeling, focusing on collagen deposition as a functional indicator of matrix synthesis. We investigated the behavior of primary human GFs cultured under 2D monolayer and 3D fibrin hydrogel conditions over time, assessing cell viability, histological characteristics, extracellular matrix production, proteolytic activity and gene expression. In addition, mechanical properties of fibrin hydrogels with and without embedded cells were analyzed. Since GFs in a 3D fibrin matrix likely resemble more closely the *in vivo* three-dimensionality with environmental clues, we hypothesized that culturing GFs within a 3D fibrin matrix would more closely recapitulate the native environment and thus promote enhanced ECM remodeling activity compared to conventional 2D culture conditions.

## Materials & methods

### Cell preparation

GFs were isolated from gingival tissue obtained from seven systemically healthy donors undergoing third molar extraction at the Department of Oral and Maxillofacial Surgery, Amsterdam UMC, Academic Centre for Dentistry Amsterdam, University of Amsterdam and Vrije University Amsterdam, Amsterdam, the Netherlands. Tissue was harvested from sites without clinical signs of gingival inflammation (probing depth ≤ 3 mm, no bleeding on probing, and no clinical attachment loss). All donors provided written informed consent. Ethical approval was obtained from the Medical Ethics Committee of Amsterdam UMC (protocol number 2016.105).

Tooth associated gingival tissue was excised using a sterile scalpel. The tissue fragments were washed twice in Dulbecco’s Modified Eagle Medium (DMEM; Gibco BRL, Paisley, Scotland) supplemented with 10% fetal calf serum (FC1; HyClone, Logan, UT, USA) and 1% PSF (100 U/mL penicillin, 100 µg/mL streptomycin, and 250 ng/mL amphotericin B; Antibiotic-Antimycotic Solution, Sigma-Aldrich, St. Louis, MO, USA). Samples were cultured in a humidified atmosphere of 5% CO_2_ at 37°C. Cells at passage 4 were used for all experiments.

GFs were isolated and expanded independently from each donor and were not pooled. All experiments were performed using primary GFs from seven independent donors, with each donor representing one biological replicate, except for the histological and stiffness measurement analyses. For each assay, measurements were performed once per donor per condition, and donor-matched comparisons between 2D and 3D culture conditions were applied throughout.

### Fibrin gel preparation

For 2D monolayer conditions, GFs were seeded in 48-well plates at a density of 3 × 10^4^ cells/well. 2D monolayers cultures were performed on standard tissue culture plastic without fibrin coating.

For 3D culture, fibrin hydrogels were prepared as described by de Jong et al [24]. Human fibrinogen (depleted of plasminogen, von Willebrand factor, and fibronectin; Enzyme Research Laboratories, South Bend, IN, USA) was reconstituted in DMEM at a concentration of 4 mg/mL. The fibrinogen solution was then mixed 1:1 either with plain DMEM (acellular control) or with a cell suspension in DMEM to achieve final concentrations of 2 mg/mL fibrinogen and 2 × 10^5^ cells/mL. Fibrin polymerization was initiated by adding human α-thrombin (1 IU/mL final concentration; Prolytix, Essex Junction, VT, USA).

The cell density for the 3D fibrin hydrogels was determined based on surface-area equivalence with the 2D monolayer. In a 48-well plate, the culture surface area is approximately 1.1 cm^2^, where 3 × 10^4^ cells were seeded per well (∼2.7 × 10^4^ cells/cm^2^). The total surface area of the fibrin gel (10 mm diameter, ∼3 mm height) was estimated at ∼ 2.5 cm^2^. Accordingly, ∼6 × 10^4^ cells would be required to maintain a comparable number of cells per available matrix surface area, corresponding to a final concentration of 2 × 10^5^ cells/mL in a 300 µL gel. This density lies within the commonly reported range (10^5^–10⁶ cells/mL) for fibroblast-mediated 3D fibrin or collagen remodeling assays, which are designed to achieve measurable matrix contraction within experimental timeframes [17], [18]. For each hydrogel, 300  µL of the fibrin mixture was dispensed per well and allowed to polymerize for 1 h at room temperature, followed by an additional hour in a humidified incubator at 37°C with 5% CO_2_. No medium was added during polymerization to avoid disruption of fibrin fibril formation.

Following polymerization, 300  µL of culture medium was added per well for both 2D and 3D conditions. The culture medium consisted of DMEM supplemented with 10% FC1, 1% PSF, and 50  µg/mL ascorbic acid (Sigma-Aldrich). The culture medium was refreshed every three days throughout the entire experiment.

### Cell viability assay

Cell viability was assessed on days 7, 14, and 21 by using the Live/Dead Viability/Cytotoxicity Kit (Invitrogen, Thermo Fisher Scientific, Life Technologies Corporation, Bend, OR, USA). A staining solution was prepared by diluting 2.5  μM calcein-AM and 10  μM ethidium homodimer-1 in phosphate-buffered saline (PBS; Thermo Fisher Scientific). Prior to staining, GFs were washed three times with PBS to remove any residual culture medium. The staining solution was subsequently added, and the samples were incubated for 1 h at room temperature in the dark. After incubation, GFs were washed again three times with PBS. Fluorescence imaging was performed at 100 × magnification using a fluorescence microscope (Leica DMIL equipped with DFC7000T camera, Leica Microsystems, Wetzlar, Germany). Images were acquired from randomly selected fields.

### Histochemistry staining

At days 7, 14, and 21, samples were first washed once with PBS, and fixed in 4% paraformaldehyde (PFA) at room temperature for 2 h. Following fixation, samples were dehydrated through a graded ethanol series (70%, 80%, 90%, 96% and 100%) and cleared in xylene. Fibrin hydrogels were then embedded in paraffin and sectioned at a thickness of 5 µm using a rotary microtome (Leitz Wetzlar Type 1212, Wetzlar, Germany). Sections were mounted on glass slides and dried overnight at 37°C.

For Hematoxylin and Eosin (H&E) staining, paraffin sections were deparaffinized in xylene, rehydrated through a descending ethanol series (100%, 96% to 50%) to distilled water, stained with Mayer’s hematoxylin solution for 5 min, rinsed in running tap water for 10 min and then counterstained with eosin Y solution for 2 min. After dehydration and clearing in xylene, coverslips were mounted using a synthetic mounting medium (DPX, Sigma-Aldrich, Saint Louis, MO, USA).

For Masson’s Trichrome (MT) staining, paraffin sections were deparaffinized and rehydrated as above. Briefly, nuclei were stained with Weigert’s iron hematoxylin solution for 3 min, rinsed, and incubated in ponceau-acid fuchsin-azophloxin solution for 12 min. After rinsing in 1% acetic acid, sections were differentiated in a phosphomolybdic acid and Orange G solution for 3 min, rinsed again in 1% acetic acid, and counterstained with light green for 5 min. Finally, sections were dehydrated, cleared in xylene, and mounted with DPX mounting medium (Sigma-Aldrich).

Stained sections were examined and imaged using a bright-field microscope (Leica DMRA, Leica Microsystems, Wetzlar, Germany).

### Gelatin zymogram

Gelatinolytic activity of secreted matrix metalloproteinases (MMPs) was evaluated using Novex Zymogram Plus (Gelatin) Protein Gels, 10%, 1.0 mm (Thermo Fisher Scientific) from conditioned media collected from 2D and 3D cultures at days 7 and 21. Equal sample volumes were mixed with non-reducing sample buffer and loaded onto the gels without boiling. Electrophoresis was performed under non-reducing conditions at 125 V in Tris-Glycine SDS running buffer. Following electrophoresis, gels were washed twice in water for 5 min at room temperature, incubated in renaturing buffer for 30 min, and then in developing buffer for 30 min at room temperature followed by overnight incubation at 37°C to permit enzymatic digestion of gelatin. Gels were subsequently stained with SimplyBlue SafeStain for 1 h and destained until clear proteolytic bands appeared against a dark blue background. Zones of gelatin degradation, corresponding to MMP activity, were visualized, imaged, and quantified by densitometric analysis using ImageJ software (NIH, USA).

### Stiffness measurement

To assess the viscoelastic properties of the 3D fibrin hydrogels, low-load compression testing (LLCT) was performed as described by Getova et al. [25], at day 1 and day 7, both in the absence and presence of cells. A stainless steel plunger (d = 2.5 mm) was used to apply 20% uniaxial compression within 1 s (strain rate = 0.2 s^−1^). The deformation was then maintained for 100 s while monitoring the stress relaxation response.

At day 1, five hydrogels per condition were analyzed, and at day 7, three hydrogels per condition were measured. For each hydrogel, measurements were taken at three random locations to determine the elastic modulus (Young’s modulus, Pa) and the half-time of relaxation (τ_1_/_2_, s). The elastic modulus was calculated from the slope of the stress–strain curve during the initial 1-second compression phase. The half-time of relaxation (τ_1_/_2_) was defined as the time point at which the stress decayed to 50% of its initial value. A strain rate of 0.2 s^−^1 (20% compression applied over 1 s) was selected to remain within the linear viscoelastic regime of fibrin hydrogels and to minimize strain-rate–dependent stiffening effects.

### RNA isolation and qPCR

Total RNA was extracted from 2D and 3D cultured cells at days 7, 14, and 21. For 3D samples, fibrin hydrogels were first snap-frozen in liquid nitrogen and mechanically disrupted using sterile RNase-free disposable pestles (Thermo Fisher Scientific). TRIzol Reagent (Thermo Fisher Scientific) was then added, and RNA isolation was performed according to the manufacturer’s instructions. For 2D samples, TRIzol was added directly to the cell monolayers. RNA concentration and purity were determined using a Synergy microplate reader (BioTek, Santa Clara, CA, USA). cDNA was synthesized using the MBI Fermentas cDNA Synthesis Kit (Vilnius, Lithuania) with both Oligo(dT)_18_ and random hexamer (D(N)_6_) primers. Gene-specific primers were designed using Primer Express software, version 2.0 (Applied Biosystems, Foster City, CA, USA), ensuring that each amplicon spanned at least one intron to prevent amplification of genomic DNA (primer sequences are listed in Table 1).

**Table 1:** Primer sequences used for quantitative RT-PCR.

**Gene**	**Sequence 5′- 3′**	**Amplicon Length (bp)**	**Ensembl Gene ID**
*HMBS*	TCCAAGCGGAGCCATGTCTG	192	ENSG00000256269
	CCTGTGGTGGACATAGCAAT		
*MKI67*	CCCTCAGCAAGCCTGAGAA	202	ENSG00000148773
	AGAGGCGTATTAGGAGGCAAG		
*ICAM1*	TGAGCAATGTGCAAGAAGATAGC	104	ENSG00000090339
	CCCGTTCTGGAGTCCAGTACA		
*ITGAV*	TACAGCAGGTCCCCAAGTCACT	100	ENSG00000138448
	AATTCAGATTCATCCCGCAGAT		
*FN1*	CACTGATTGCACTTCTGAGG	179	ENSG00000115414
	CCATGTCATGCTGCTTATCC		
*COL1A1*	TCCGGCTCCTGCTCCTCTTA	336	ENSG00000108821
	GGCCAGTGTCTCCCTTG		
*COL3A1*	GTCAGTCCTATGCGGATAGA	206	ENSG00000168542
	AGGTCCTTGACCATTAGGAG		
*PLAU*	TGGAACTCTGCCACTGTCCTT	100	ENSG00000122861
	TTGTCTGGGTTCCTGCAGTAATT		
*PLAT*	CGGACTGGACGGAGTGTGA	105	ENSG00000104368
	TGGATGGGTACAGTCTGACATGA		
*TGFB1*	CTACTACGCCAAGGAGGTCA	199	ENSG00000105329
	CACGTGCTGCTCCACTTT		
*ACTA2*	GAGTCTGCTGGCATCCATGA	220	ENSG00000107796
	CACCGATCCAGACAGAGTA		
*HMBS*, hydroxymethylbilane synthase (encoding for porphobilinogen deaminase; PBGD); *MKI67*, antigen Kiel (KI-67); *ICAM1*, intercellular adhesion molecule 1 (ICAM-1); *ITGAV*, integrin subunit alpha V (integrin α_V_); *FN1*, fibronectin 1; *COL1A1*, collagen type I alpha 1 chain; *COL3A1*, collagen type III alpha 1 chain; *PLAU*, plasminogen activator, urokinase (u-PA); *PLAT*, plasminogen activator, tissue type (t-PA); *TGFB1*, transforming growth factor beta-1 (TGF-β1); *ACTA2*, actin alpha 2, smooth muscle (α-SMA). For each gene, the first sequence represents the forward primer, the second sequence the reverse primer**.**			

qPCR was performed on a LightCycler 480 system (Roche, Basel, Switzerland). The thermal cycling protocol included an initial DNA polymerase activation step at 94°C for 10 min, followed by 40 cycles of a two-step amplification: denaturation at 95°C for 30 s, and combined annealing/extension at 60°C for 1 min. A melting curve analysis was performed at the end of the run to confirm the specificity of the amplification and the absence of nonspecific products.

The expression of each gene of interest was normalized to the expression of the housekeeping gene *HMBS*, encoding for PBGD, which was confirmed to remain stable under all experimental conditions. Relative gene expression was calculated using the ΔCt method (Ct_gene of interest_- Ct_HMBS_), and results were expressed as 2 ^–(ΔCt)^.

### Statistical analysis

Statistical analyses were performed using SPSS software (version 30.0; IBM, New York, USA), and graphs were generated using GraphPad Prism (version 10.2.3; GraphPad Software, La Jolla, CA, USA). Data distribution was assessed using the Shapiro–Wilk test, which indicated normal distribution for gelatin zymography data, and non-normal distribution for both qPCR, stiffness and relaxation measurements.

For gelatin zymography data, changes in the MMP activity over time were analyzed using paired sample *t*-test for pairwise comparisons between 2D and 3D conditions at each time point, as well as between specific time points. For the qPCR data, changes in gene expression over time were analyzed using Friedman’s test with Dunn’s post hoc multiple comparisons for between-group analysis. Pairwise comparisons between 2D and 3D conditions at each time point were conducted using the Wilcoxon signed-rank test. For stiffness and relaxation measurements, Wilcoxon signed-rank test used for pairwise comparisons between 2D and 3D conditions at each time point and between specific time points. A p-value ≤ 0.05 was considered statistically significant.

## Results

### GFs maintain viability and exhibit distinct morphology in 3D fibrin hydrogels

**Fig. 1:**
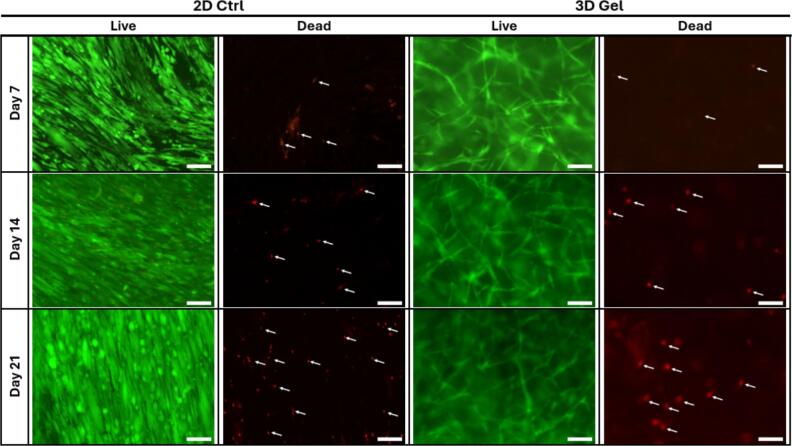
**The viability of gingival fibroblasts (GFs) cultured in 2D monolayers (2D Ctrl) and 3D fibrin hydrogels (3D Gel) over time.** In both conditions, GFs maintained viability, and in 3D fibrin hydrogels, they displayed distinct morphology with elongated and branched protrusions compared to 2D monolayers. Fluorescence images of live cells (green) and dead cells (red). White arrows point out dead cells. Live-dead staining was performed on all seven donors. Scale bar: 250 μm. (For interpretation of the references to colour in this figure legend, the reader is referred to the web version of this article.)

The viability of GFs cultured in 2D monolayers and 3D fibrin hydrogels was assessed at days 7, 14, and 21 using Live/Dead staining. In both culture conditions, the majority of the cells exhibited strong green fluorescence, indicating high viability throughout the culture period. Only a limited number of red fluorescent cells, indicative of cell death, were observed at any time point in both culture conditions, showing a gradual increase from day 7 to day 21 as pointed out with white arrows in Fig. 1.

Notably, GFs in 3D fibrin hydrogels displayed a distinct morphology characterized by elongated shapes and branched cellular protrusions extending within the fibrin network, whereas GFs in 2D monolayers showed the spindle-shaped morphology. Representative fluorescence images showing live (green) and dead (red) cells at each time point are presented in Fig. 1.

### GFs remodel 3D fibrin hydrogels over time

Histological analysis confirmed that the 3D fibrin hydrogels were successfully formed and structurally maintained throughout the 21-day culture period. H&E staining revealed that GFs were evenly distributed within the 3D hydrogels and retained their presence over time. Consistent with the Live/Dead assay, GFs exhibited an elongated, web like morphology suggestive of active spreading and adaptation to the 3D environment.

**Fig. 2:**
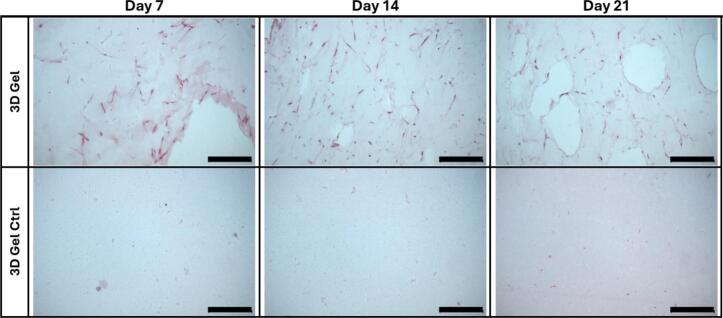
**Hematoxylin and eosin (H&E) staining of 3D fibrin hydrogels with and without GFs. A-C)** Upper row: GFs seeded in 3D fibrin hydrogels (3D Gel) at day 7, 14, and 21. Cells were visible throughout the matrix, displaying elongated morphology and progressive matrix remodeling, with noticeable voids forming by day 21. **D-F)** Lower row: Acellular control gels (3D Gel Ctrl) showed intact fibrin structure without evidence of cell presence or degradation. Representative gels (n = 2) were analyzed in detail. Scale bar: 250 μm.

By day 21, acellular regions or voids appeared adjacent to GF-populated areas, suggesting local degradation of the fibrin matrix. In contrast, control gels without seeded cells remained structurally intact, with no visible signs of degradation and no cellular presence, suggesting that matrix remodeling was GF-mediated. Representative histological images are presented in Fig. 2.

### GFs promote collagen deposition in 3D fibrin hydrogels

Masson’s Trichrome staining demonstrated that GFs actively remodeled the 3D fibrin hydrogels by producing collagen. Collagen deposition was evident by the progressive appearance of blue/green staining within the hydrogel, increasing over time from day 7 to day 21. This staining pattern indicates early connective tissue formation driven by cellular activity.

**Fig. 3:**
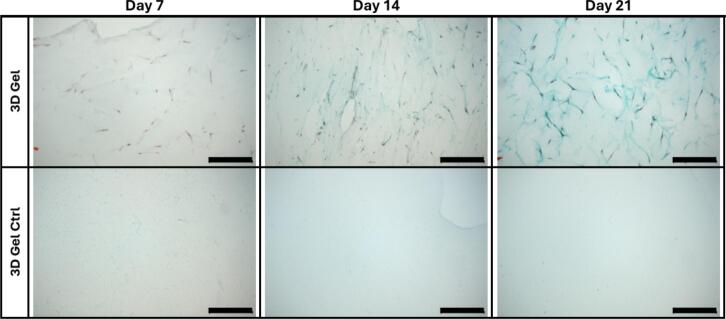
**Masson’s Trichrome (MT) staining of 3D fibrin hydrogels with and without GFs. A-C)** Upper row: GFs seeded in 3D fibrin hydrogels (3D Gel) at day 7, 14, and 21 showed progressive blue/green staining, indicating collagen deposition and matrix remodeling. **D-F)** Lower row: Acellular controls (3D Gel Ctrl) showed no collagen staining or structural changes over time, confirming the absence of new extracellular matrix production. Representative gels (n = 2) were analyzed in detail. Scale bar: 250 μm. (For interpretation of the references to colour in this figure legend, the reader is referred to the web version of this article.)

In contrast, control hydrogels without cells exhibited no detectable collagen staining and remained structurally unchanged, confirming that matrix remodeling and *de novo* ECM synthesis occurred only in the presence of GFs. Representative images are shown in Fig. 3.

### MMP-2 activity increased over time

**Fig. 4:**
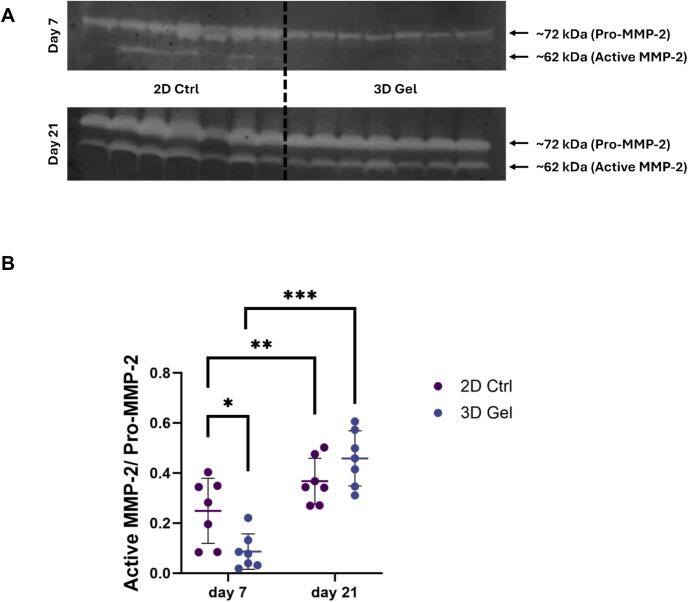
**Gelatin zymography analysis of MMP-2 activity in GFs cultured in 2D monolayers (2D Ctrl) and 3D fibrin hydrogels (3D Gel). A)** Representative zymogram showing clear bands corresponding to the pro- (∼72 kDa) and active (∼62 kDa) forms of MMP-2 at days 7 and 21. **B)** Quantification of MMP-2 activity expressed as the ratio of active MMP-2 to pro-MMP-2. At day 7, MMP-2 activity was higher in 2D monolayers than 3D fibrin hydrogels (p < 0.05). By day 21, MMP-2 activity increased in both culture systems, with no significant difference between conditions (p > 0.05). A significant time-dependent increase in MMP-2 activity was observed in both 2D and 3D cultures (p < 0.01 and p < 0.001, respectively). (Paired sample *t*-test for pairwise comparisons). Data are presented as mean ± SD of seven independent donors (n = 7), each measured once per condition. * *p* < 0.05, ** *p* < 0.01, *** *p* < 0.001.

To assess the proteolytic activity of the GFs in 2D monolayers and 3D fibrin hydrogels, gelatin zymography of tissue culture supernatants was performed at day 7 and day 21. Clear bands corresponding to the pro- and active forms of MMP-2 were detected at approximately 72 kDa and 62 kDa, respectively (Fig. 4A). MMP-2 activity was quantified as the ratio of active MMP-2 to pro-MMP-2.

At day 7, MMP-2 activity was significantly higher in 2D monolayers compared to 3D fibrin hydrogels (*p* < 0.05; Fig. 4B). By day 21, MMP-2 had markedly activity increased in both conditions, and the difference between 2D and 3D cultures was no longer significant (*p* > 0.05; Fig. 4B). When comparing time points within each condition, MMP-2 activity increased significantly from day 7 to day 21 in both 2D monolayers and 3D fibrin hydrogels (*p* < 0.01 and *p* < 0.001, respectively; Fig. 4B). These results indicate that MMP-2 activity progressively increased over time, suggesting ongoing matrix remodeling mediated by GFs.

### Stiffness and relaxation properties of 3D fibrin hydrogels remained stable over time

**Fig. 5:**
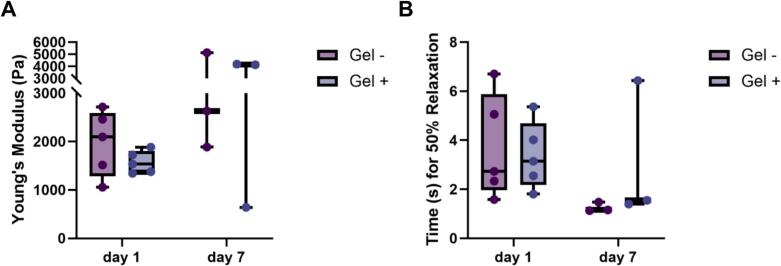
**Stiffness and stress relaxation properties of 3D fibrin hydrogels. A)** Elastic modulus (Young’s modulus, Pa) of 3D fibrin hydrogels measured by low-load compression testing (LLCT) at day 1 and day 7, with (Gel + ) or without (Gel–) embedded gingival fibroblasts (GFs). Hydrogels were compressed by 20% within 1 s, and force response was recorded over 100 s. Although a slight increase in stiffness was observed at day 7, this difference was not statistically significant (p > 0.05). **B)** Half-time of stress relaxation (τ_1_/_2_, s), representing the time required for 50% stress decay. A slight, non-significant decrease in τ_1_/_2_ was observed at day 7, suggesting increased matrix compliance over time (Wilcoxon signed-rank test for pairwise comparisons, p > 0.05). Data are presented as median and range. n = 5 at day 1, n = 3 at day 7.

To examine temporal changes in the mechanical behavior of 3D fibrin hydrogels, LLCT was performed at day 1 and day 7 in hydrogels with or without embedded GFs. The presence of cells did not significantly influence the stiffness of the hydrogels at either time point (*p* > 0.05; Fig. 5A). Although a slight increase in stiffness was observed at day 7 compared to day 1, this difference was not statistically significant. Overall, the elastic modulus of the fibrin matrices remained stable over time, independent of cell presence.

Stress relaxation behavior was also analyzed to assess viscoelastic properties. The half-time of relaxation (τ_1_/_2_), representing the time required for 50% stress decay, showed a slight but non-significant decrease from day 1 to day 7 (*p* > 0.05; Fig. 5B). This trend suggests that prolonged incubation may increase matrix compliance, making the hydrogels more responsive to cellular contractile forces.

### Gene expression profile of GFs differs in 2D and 3D cultures

To investigate the dynamic gene expression changes of GFs in 2D monolayers and in 3D fibrin hydrogels, a panel of genes related to proliferation, cell attachment, ECM production and remodeling, matrix degradation, and myofibroblast differentiation was analyzed using RT-qPCR. Comparisons were made between 2D monolayer cultures and 3D fibrin hydrogels at days 7, 14, and 21.

**Fig. 6:**
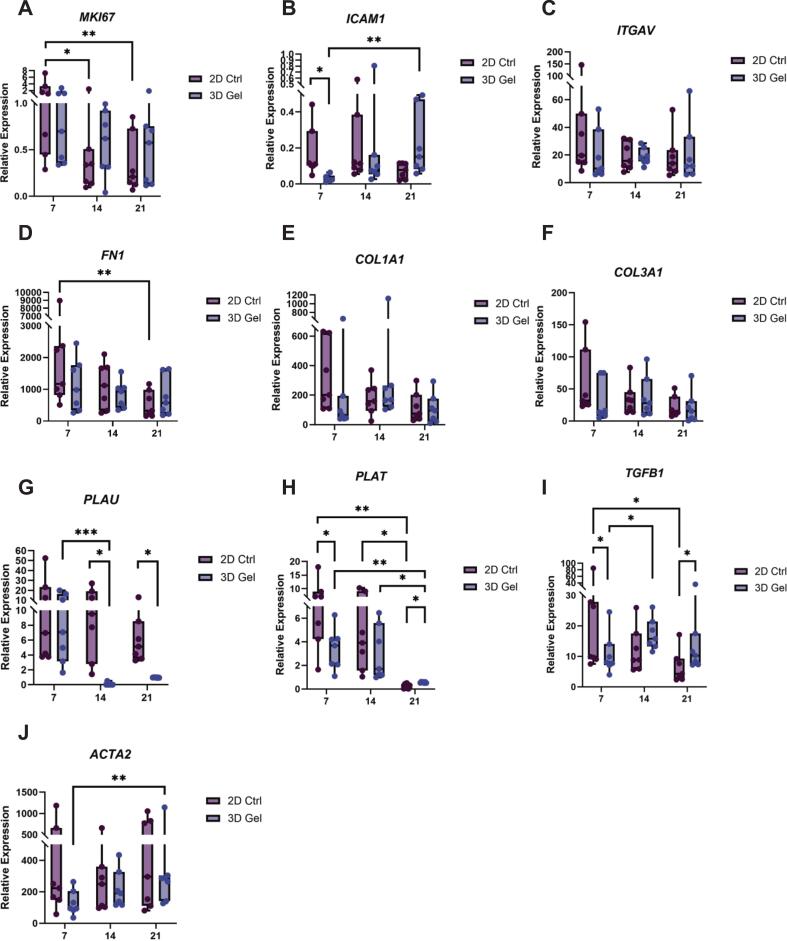
**Gene expression analysis of GFs cultured in 2D monolayers (2D Ctrl) and 3D fibrin hydrogels (3D Gel) over time.** Gene expression levels were measured at days 7, 14, and 21. **A)***MKI67* (KI-67), **B)***ICAM1* (ICAM-1), **(C)***ITGAV* (αv Integrin), **D)***FN1* (Fibronectin), **E)***COL1A1*, **F)***COL3A1*, **G)***PLAU* (u-PA), **H)***PLAT* (t-PA), **I)***TGFB1* (TGF-β1), and **J)***ACTA2* (α-SMA). Boxplots show median, range, and individual data points. Each data point represents one donor (biological replicate; n = 7). * *p* < 0.05, ** *p* < 0.01, *** *p* < 0.001.

Expression levels of the proliferation marker KI-67 (*MKI67*; Fig. 6A) showed significant decrease in 2D monolayers between day 7 and both day 14 (*p* < 0.05) and day 21 (*p* < 0.01), whereas KI-67 remained relatively constant in the 3D cultures, suggesting that proliferation took place over a longer time. However, no significant differences were observed between 2D and 3D conditions at any time point (*p* > 0.05). ICAM-1, an important molecule involved in both cell–cell and cell–matrix interactions, showed significantly higher expression in 2D conditions compared to 3D fibrin hydrogel at day 7 (*p* < 0.05; Fig. 6B). In 3D hydrogels, ICAM-1 expression increased significantly between day 7 and day 21 (*p* < 0.01; Fig. 6B). Since proper cell attachment is essential for signaling between cells and their environment, we also measured the expression levels of α_V_ integrin (*ITGAV*; Fig. 6C). Overall, expression levels were comparable over time in both conditions and showed no significant differences (p > 0.05).

Since GFs are key cells for oral wound healing, and as they deposit and turnover ECM, we measured gene levels of several ECM proteins, one of which was fibronectin, which is an important linker protein that connects collagen type I to cell-surface integrins and facilitates fibroblast migration within the matrix [26]. Among the ECM-related genes, fibronectin (*FN1*; Fig. 6D) displayed highest expression levels. Although no statistically significant difference was found between 2D and 3D conditions at any timepoint (*p* > 0.05), fibronectin levels in 2D conditions significantly decreased over time, particularly, between day 7 and 21(*p* < 0.01). During wound healing, the initial fibrin-based provisional matrix is gradually replaced by a collagen-rich matrix synthesized by fibroblasts, in which collagen type III serves as an early structural component that is later remodeled into collagen type I fibers [10]. Expression levels of both collagen type 1 (*COL1A1*; Fig. 6E) and collagen type 3 (*COL3A1*; Fig. 6F), were comparable over time and didn’t show any statistically significant difference between 2D and 3D conditions at any timepoint (*p* > 0.05).

As fibrin acts as a provisional matrix at the early stages of wound healing, and is being degraded and replaced by native ECM, it is important to measure the genes that are involved in the matrix degradation phase. Therefore, to assess the fibrinolytic activity, we measured the gene expression levels of two proteases that are responsible for fibrin degradation; u-PA and t-PA. At day 7, u-PA (*PLAU*; Fig. 6G) expression levels didn’t show any significant difference between 2D and 3D conditions (*p* > 0.05). However, at both day 14 and 21, u-PA levels in 2D conditions were significantly higher than 3D conditions (*p* < 0.05 and *p* < 0.05, respectively), suggesting a more anabolic stage in later stage 3D cultures. In addition, a significant downregulation was seen in 3D conditions between day 7 and day 14 (*p* < 0.001).

Similarly, t-PA (*PLAT*; Fig. 6H) expression declined over time in both conditions. In 2D monolayers, its levels significantly decreased from day 7 to day 14 (*p* < 0.05), and from day 14 to day 21 (*p* < 0.05). In 3D hydrogels, t-PA expression also decreased significantly between day 7 and day 21(*p* < 0.01), and between day 14 and day 21(*p* < 0.05). Notably, at day 21, t-PA levels was higher in 3D hydrogels than 2D monolayers (*p* < 0.05).

TGF-β1 (*TGFB1*; Fig. 6I) is a multifunctional cytokine that plays a central role in regulating fibroblast behavior. It promotes fibroblast migration, stimulates the synthesis of key ECM components such as fibronectin, collagen type I, and collagen type III, and induces fibroblast differentiation into myofibroblasts while enhancing *MMP-2* production [27]. Consistent with its pivotal role in wound healing, TGF-β1 expression showed higher expression in 2D conditions than 3D hydrogels at day 7 (*p* < 0.05). In 2D monolayers, TGF-β1 expression decreased significantly from day 7 to day 21 (*p* < 0.05), while in 3D hydrogels, it increased from day 7 to day 14 (*p* < 0.05), and it was higher in 3D fibrin hydrogels than 2D monolayer at day 21 (*p* < 0.05). These findings reflect dynamic regulation of TGF-β1 in response to microenvironmental cues.

Lastly, expression of α-SMA (*ACTA2*; Fig. 6J), a hallmark of myofibroblast differentiation and fibrin contraction, was significantly upregulated in 3D hydrogels from day 7 to day 21 (*p* < 0.01). During normal wound healing, TGF-β1 plays a central role in driving fibroblast-to-myofibroblast transition and in promoting fibronectin synthesis [28], [29]. This process is closely associated with the activation of focal adhesion kinase (FAK), which is essential for myofibroblast differentiation and is characterized by the formation of α-SMA-containing stress fibers[29]. These cytoskeletal changes enhance cellular contractility and are accompanied by increased production of ECM proteins such as collagen type I, leading to progressive alterations in the mechanical and structural properties of the surrounding matrix [28]. Collectively, our findings indicate a temporal shift toward a myofibroblast-like phenotype within the 3D fibrin hydrogels, consistent with active matrix remodeling and maturation.

## Discussion

Understanding the reciprocal interactions between cells and the ECM is essential for elucidating the cellular and molecular mechanisms that govern periodontal and *peri*-implant wound healing and tissue regeneration. In the present study, we demonstrated that 3D fibrin hydrogels provide a biologically active and spatially relevant environment that more closely mimics *in vivo* conditions compared with conventional 2D monolayer cultures. GFs cultured in 3D fibrin hydrogels maintained viable and displayed distinct morphological phenotype characterized by elongated cell bodies and branched protrusions, in contrast to the flattened, spindle-shaped morphology observed in 2D cultures. Moreover, the 3D fibrin hydrogels allowed the visualization of ECM remodeling over time, including fibrin degradation and the deposition of newly synthesized ECM components such as collagen, as confirmed by Hematoxylin & Eosin, and Masson’s Trichrome staining. These findings indicate that fibrin-based 3D systems provide a physiologically relevant microenvironment for studying fibroblast behavior and wound healing-related processes. Furthermore, this 3D fibrin network establishes a robust platform for future studies aiming to model more complex aspects of periodontitis, including the roles of inflammatory cytokines, leukocytes, and bacterial components.

Although 2D culture systems have long been widely used for studying cellular responses under controlled conditions, they fail to recapitulate the complexity of *in vivo* tissues, particularly regarding cell–cell and cell–matrix interactions. In contrast, 3D culture systems more accurately reproduce the spatial organization, mechanical cues, and diffusion gradients that cells experience in native tissues, which ultimately influence proliferation, differentiation, and mechanosensing [20]. Additionally, 3D cell culture models allow for the use of histological methods to assess intercellular relationships and ECM deposition [30]. In this line, histological analyses of our 3D fibrin hydrogels revealed progressive ECM remodeling over time, with collagen deposition in close proximity to fibroblasts, consistent with findings by San Martin et al. who reported complete replacement of fibrin scaffolds by human oral fibroblasts that synthesized ECM components such as collagen type I and type III [14]. In 2D cultures, *MKI67* (KI-67) gene expression decreased over time, which can be attributed to contact inhibition, a well-known phenomenon whereby proliferating cells cease division upon reaching confluence [31]. This process represents normal cell cycle arrest rather than protein depletion, consistent with the plateau phase of monolayer cultures. In contrast, in 3D fibrin matrices, fibroblasts remain surrounded by available space and a supportive ECM network, allowing sustained cell activity and migration throughout the culture period.

Integrins are transmembrane heterodimeric glycoproteins composed of α and β subunits that mediate cell adhesion to specific ECM ligands [32]. Integrin-mediated signaling regulates a variety of cellular processes including adhesion, migration, proliferation, and matrix contraction, all of which are essential for wound healing and tissue regeneration [33], [34]. Because fibrin acts as a provisional matrix during early wound healing, fibroblast adhesion to fibrin is a prerequisite for subsequent remodeling [35]. Farrell et al. demonstrated that fibroblast adhesion to fibrinogen is both time- and dose-dependent and occurs via RGD−dependent and −independent mechanisms involving αV integrins and ICAM-1, respectively [36]. This mechanism corresponds with our findings, where *ICAM1* expression increased in 3D fibrin hydrogels between day 7 and day 21. This increase likely reflects progressive cell proliferation, spreading, and cell–cell connectivity within the fibrin network as the culture matured. Meanwhile, *ITGAV* (αV integrin) expression remained relatively constant between 2D and 3D cultures over time, suggesting that αV integrin expression in GFs is constitutive. Indeed, functional modulation of αV integrins occurs predominantly via conformational activation, heterodimer pairing, and cytoskeletal coupling rather than transcriptional regulation [37]. Furthermore, the addition of ascorbic acid in the culture medium, which enhances collagen biosynthesis and ECM maturation, may have indirectly altered integrin engagement without modifying transcriptional changes [38].

As wound healing progresses, fibroblasts replace the provisional fibrin matrix with a new collagen-rich ECM [5]. Fibroblasts are responsible for synthesizing both collagen and fibronectin, which together form the scaffold necessary for cell migration and tissue maturation [39], [40]. In our study, *FN1* (fibronectin) expression was markedly higher than the other ECM-related genes analyzed, approximately 10-fold greater than collagen genes. This finding aligns with previous studies showing that fibronectin is critical for fibroblast migration within fibrin clots [26]. Greiling et al. demonstrated that the absence of fibronectin reduced fibroblast migration into fibrin gels by nearly 80%, emphasizing the essential role of fibronectin as a cell−adhesive linker that facilitates integration with other ECM proteins [26]. Thus, our data indicate the prominent role of fibronectin in promoting fibroblast migration and matrix organization during the remodeling process.

TGF-β1 is a key growth factor regulating fibroblast activity, stimulating proliferation, collagen synthesis like type I and III, and myofibroblast differentiation through an autocrine feedback loop [5], [27]. Retamal et al. showed that TGF-β1 promoted human gingival fibroblast differentiation into α-SMA-positive myofibroblasts with enhanced collagen and fibronectin production, and actin cytoskeletal organization [41]. Additionally, the expression of fibroblast integrins, such as αVβ3 and αVβ5, stimulates TGF-β1 synthesis, which is associated with myofibroblast formation [42]. In our study, *TGFB1* expression increased significantly between day 7 and day 14 and was higher in 3D fibrin hydrogels compared to 2D monolayers. This correlated with an increase in *ACTA2* (α-SMA) expression between days 7 and 21, indicating that fibroblasts in the 3D fibrin hydrogels were undergoing myofibroblast-like differentiation under the influence of TGF-β1 signaling. Furthermore, *COL1A1* and *COL3A1* were also upregulated during this period, suggesting enhanced collagen deposition and matrix maturation. The relatively high gene expression of these ECM components in 2D cultures may be attributed to the extremely stiff substrate (1–3 GPa) of standard tissue culture plastic, which can induce fibroblast activation through mechanotransduction, mimicking myofibroblast-like behavior[43].

As wound healing advances, ECM composition transitions from a fibronectin− and collagen type III–rich matrix to one dominated by collagen type I, which provides mechanical strength and stability [39], [44]. This remodeling is driven primarily by matrix metalloproteinases (MMPs) [3]. MMP-2, a fibroblast-secreted gelatinase, degrades fibronectin and denatured collagens, thus facilitating new matrix deposition [45]. Our zymography results demonstrated a significant increase in MMP-2 activity over time in both 2D and 3D conditions. Initially, MMP-2 activity was higher in 2D cultures at day 7, possibly due to higher *TGFB1* expression. However, by day 21, MMP-2 activity increased markedly in both conditions, with no significant difference between them. This progressive activation of MMP-2 supports ongoing matrix remodeling. Kobayashi et al. similarly showed that TGF-β1 enhances MMP-2 production in dermal fibroblasts, consistent with the elevated *TGFB1* expression observed here [46]. Miller et al. demonstrated that, after seven days of culture, the activated form of MMP-2 was higher than the pro-form of the enzyme in both dermal and gingival fibroblasts [47]. While the active/pro-MMP-2 ratio in our study did not exceed 1, the upward trend indicates increasing enzymatic activation. The relatively lower MMP-2 activity compared with collagen lattice systems, as shown by Miller et al., likely results from the limited substrate availability in fibrin matrices, where fibroblasts are only beginning to produce new ECM.

The fibrinolytic system also contributes to matrix remodeling by activating MMPs and releasing latent growth factors such as TGF-β1 [48], [49]. Both *PLAU* (u-PA) and *PLAT* (t-PA) facilitate fibrin degradation and dermal fibroblast migration during early human skin wound healing [50]. Consistent with this, our results showed decreased *PLAT* expression at day 21 and reduced *PLAU* expression in 3D fibrin hydrogels at days 14 and 21. This downregulation likely reflects temporal regulation of fibrinolytic enzymes and the inhibitory effect of TGF-β1, where their early activity promotes initial matrix breakdown and cell invasion, followed by suppression once the matrix shifts toward collagen deposition. Sarajlic et al. reported similar findings, showing that TGF-β1 exposure reduced plasminogen activation in periodontal ligament and gingival fibroblasts, primarily via u-PA inhibition [51]. The temporal relationship between plasminogen activators and MMPs has been demonstrated in a mouse dermal wound model, where u-PA and t-PA activity peaked prior to MMP-2 activation, and remained elevated during scar maturation [49]. As shown by Wang et al. in a transcriptomic analysis of human gingiva during wound healing, MMP-2 was among the MMP genes that were significantly upregulated [52]. All of these results are consistent with our observation of progressively increasing MMP-2 activity over time.

*In vivo* gingival wound healing, such as after tooth extraction or periodontal surgery, is initiated by the formation of a provisional fibrin-rich matrix that supports fibroblast migration, granulation tissue formation, and subsequent ECM remodeling [53], [54]. Histological studies of canine gingival and periodontal wounds show early fibroblast infiltration into this fibrin scaffold, followed by progressive fibrin degradation and replacement with collagen-rich connective tissue, predominantly collagen types III and I [53], [54]. Consistent with these observations, GFs cultured in 3D fibrin hydrogels in the present study exhibited elongated, interconnected morphologies, localized matrix voids consistent with fibrinolysis, and increasing collagen deposition over time, resembling key histological features of gingival connective tissue remodeling observed *ex vivo*.

At the molecular level, gingival and periodontal wound healing *in vivo* is characterized by dynamic regulation of profibrotic and matrix-remodeling mediators, including TGFB1, ACTA2, and MMP2, particularly during granulation tissue formation and remodeling phases [27], [55]. In line with this, GFs in our 3D fibrin cultures showed a temporal increase in TGFB1 expression, accompanied by upregulation of ACTA2 and progressive activation of MMP-2. Together, these histological and transcriptional findings support the physiological relevance of the 3D fibrin model as a simplified yet biologically convenient platform for studying fibroblast-mediated gingival connective tissue repair.

The mechanical properties of fibrin are crucial for its biological function and are influenced by multiple factors including fibrinogen and thrombin concentrations, crosslinking density, pH, and calcium (Ca^2+^) content, all of which affect fiber thickness, porosity, and branching, and subsequently fibrin stiffness and its resistance to fibrinolysis [19]. To maintain physiological relevance, we prepared fibrin hydrogels using plasma-level fibrinogen concentrations (∼2.5 g/L) [56]. Our mechanical measurements revealed no significant changes in stiffness or relaxation properties between day 1 and day 7, nor between cell-seeded and acellular gels. This result contrasts with the findings of Jansen et al., who reported that human CCL-224 fibroblast contraction increased fibrin stiffness through myosin-II–mediated traction forces [17]. This discrepancy may stem from methodological differences and gel formulation. Jansen et al. used oscillatory shear rheometry on constrained gels, where fibroblast contractile prestress induced strain stiffening in fibrin matrix, whereas, in our setup, low-load compression test (LLCT) evaluated the bulk viscoelastic response of free-standing gels. Fibroblast-generated stresses (10^2^ Pa) are small relative to the kPa-scale compressive modulus of fibrin gels and thus unlikely to significantly alter macroscopic stiffness. Consequently, while we did not detect bulk stiffening, localized remodeling and fiber alignment likely occurred at the microscale. An additional explanation for the slight but non-significant increase in stiffness observed at day 7 may involve the effects of calcium on fibrin polymerization. Although Ca^2+^ binding does not influence the thrombin-mediated conversion of fibrinogen to fibrin, it modulates polymerization by promoting lateral aggregation and the formation of thicker fibers, thereby increasing matrix stiffness [19], [57] In our culture system, the only calcium source was the culture medium, which contains relatively low Ca^2+^ levels. A gradual calcium accumulation during the culture period may have subtly influenced fibrin architecture and mechanical properties.

The relatively wide range of stiffness values observed at day 7 likely reflects biological and structural heterogeneity inherent to cell-remodeled fibrin hydrogels. Fibroblast-mediated fibrin degradation, local collagen deposition, and fiber realignment occur in a spatially heterogeneous manner, resulting in microscale variations that may not uniformly affect bulk mechanical properties [17], [18]. In addition, donor-dependent differences in fibroblast contractility and remodeling capacity may further contribute to variability. Importantly, despite this spread, no consistent or statistically significant changes in bulk stiffness were detected, indicating that matrix remodeling predominantly occurred at the microscale rather than inducing global stiffening within the timeframe studied.

Finally, fibrin networks exhibit pronounced strain-rate– and strain-amplitude–dependent mechanical behavior. At higher deformation rates, fibrin undergoes strain stiffening due to fiber alignment and nonlinear elasticity [15], [19]. The selected strain rate in the present study therefore reflects quasi-static bulk mechanical properties rather than dynamic or cytoskeleton-driven responses. While this approach allows robust comparison between experimental conditions, future studies incorporating frequency- or rate-dependent testing may provide additional insight into fibrin viscoelasticity during active cellular remodeling.

This study is not without limitations. The 2D culture plates were not fibrin-coated, which would have allowed a more direct and comparable assessment of fibroblast behavior between culture systems, as demonstrated by Hakkinen et al. [58]. While standard tissue culture plastic represents a widely used reference condition, fibrin-coated 2D surfaces would have allowed further dissection of ligand density effects independent of culture dimensionality. Moreover, immunohistochemical analyses were not performed to identify specific ECM proteins, which would have provided further insight into the composition of the newly deposited matrix. In addition, cell viability was assessed qualitatively, as accurate quantitative viability analysis was technically challenging due to the thickness and optical limitations of the 3D fibrin hydrogels; nevertheless, qualitative Live/Dead staining consistently indicated high cell viability throughout the 21-day culture period. Finally, extending the mechanical testing beyond seven days would be valuable to capture potential long-term changes in matrix maturation and stiffening.

## Conclusion

In conclusion, this study demonstrates that 3D fibrin hydrogels provide a more physiologically relevant environment than conventional 2D monolayers for investigating gingival fibroblast behavior and ECM remodeling. The 3D system promoted *in vivo*-like cell morphology, collagen deposition, temporally regulated expression of remodeling-related genes, and progressive activation of MMP-2, all key features of wound healing. These findings underscore the potential of fibrin-based 3D culture systems as translational platforms for studying periodontal and *peri*-implant soft tissue regeneration and for designing therapeutic strategies that emulate native tissue architecture and mechanical cues.

## CRediT authorship contribution statement

**Merve Ceylan:** Writing – original draft, Visualization, Methodology, Formal analysis, Data curation, Conceptualization. **Marja L. Laine:** Writing – review & editing, Supervision, Methodology, Conceptualization. **Behrouz Zandieh Doulabi:** Data curation, Writing – review & editing. **Hans J.A.M. Korfage:** Data curation, Writing – review & editing. **René J.B. Dijkstra:** Data curation, Writing – review & editing. **Teun J. de Vries:** Writing – review & editing, Supervision, Methodology, Formal analysis, Conceptualization. **Ton Schoenmaker:** Writing – review & editing, Supervision, Methodology, Data curation, Conceptualization.

## Declaration of competing interest

The authors declare that they have no known competing financial interests or personal relationships that could have appeared to influence the work reported in this paper.

## Data Availability

Data will be made available on request.
